# The high-intensity option of the SANS diffractometer KWS-2 at JCNS – characterization and performance of the new multi-megahertz detection system^1^

**DOI:** 10.1107/S1600576718004132

**Published:** 2018-03-28

**Authors:** Judith Elizabeth Houston, Georg Brandl, Matthias Drochner, Günter Kemmerling, Ralf Engels, Aristeidis Papagiannopoulos, Mona Sarter, Andreas Stadler, Aurel Radulescu

**Affiliations:** a Jülich Centre for Neutron Science at Maier Leibnitz-Zentrum, Forschungszentrum Jülich GmbH, Lichtenbergstrasse 1, Garching 85747, Germany; b Jülich Centre for Neutron Science (JCNS-2), Forschungszentrum Jülich GmbH, Wilhelm-Johnen-Strasse, Jülich 52428, Germany; c Theoretical and Physical Chemistry Institute, National Hellenic Research Foundation, 48 Vass. Constantinou Avenue, Athens 11635, Greece; d Institute of Physics (IA), Rheinisch Westfälische Technische Hochschule Aachen (RWTH Aachen), Templergraben 55, Aachen 52056, Germany; e Jülich Centre for Neutron Science (JCNS-1), Institute for Complex Systems (ICS), Forschungszentrum Jülich GmbH, Wilhelm-Johnen-Strasse, Jülich 52428, Germany

**Keywords:** small-angle neutron scattering (SANS), neutron detectors, mesoscale structures, event-mode capability

## Abstract

A new detection system based on an array of ^3^He tubes and innovative fast detection electronics has been installed on the high-intensity small-angle neutron scattering (SANS) diffractometer KWS-2 operated by the Jülich Centre for Neutron Science (JCNS) at the Heinz Meier-Leibnitz Zentrum in Garching, Germany. The new detection system is composed of 18 eight-pack modules of ^3^He tubes that work independently of one another (each unit has its own processor and electronics). To improve the read-out characteristics and reduce the noise, the detection electronics are mounted in a closed case on the rear of the ^3^He tubes’ frame. The tubes’ efficiency is about 85% (for λ = 5 Å) and the resolution slightly better than 8 mm. The new detection system is characterized by a dead-time constant of 3.3 µs per tube and an overall count rate as high as 6 MHz at 10% dead-time loss. Compared with the old detector this is an improvement by a factor of 60. The much higher count rate will shorten the measurement times and thus increase the number of experiments possible in a given time period by the optimal use of the high flux of up to 2 × 10^8^ n cm^−2^ s^−1^ at the sample position. Combined with the event-mode operation capability, this will enable new scientific opportunities in the field of structural investigations of small soft-matter and biological systems. The implementation of the detector in the high-intensity concept on KWS-2, its characterization and its performance based on test experiments are reported in this paper.

## Abstract 2

A new detection system based on an array of ^3^He tubes and innovative fast detection electronics has been installed on the small-angle neutron scattering (SANS) diffractometer KWS-2 at the Jülich Centre for Neutron Science (JCNS), Germany. The high counting rates that can be detected and the event-mode capability will enable new scientific opportunities in the field of structural investigation of small soft-matter and biological systems.

## 1. Introduction   

The small-angle neutron scattering (SANS) diffractometer KWS-2, operated by the Jülich Centre for Neutron Science (JCNS) at the Heinz Maier-Leibnitz Zentrum (MLZ) in Garching, Germany, is dedicated to the investigation of mesoscopic multi-scale structures and structural changes due to rapid kinetic processes in soft condensed matter and biophysical systems. Following demands from the user community, it has recently been considerably upgraded with respect to the intensity on the sample, the instrumental resolution and the minimum scattering variable *Q*_min_ (Radulescu *et al.*, 2015 ▸; Radulescu, Szekely *et al.*, 2016 ▸) [*Q* = (4π/λ)sinθ is the momentum transfer, where λ is the neutron wavelength and 2θ is the scattering angle]. In the high-intensity mode, an intensity gain of up to 12 times compared with the conventional pinhole mode for the same resolution can be achieved with lenses, on the basis of increasing the sample size (Radulescu, Szekely *et al.*, 2016 ▸; Dahdal *et al.*, 2014 ▸). In the tunable resolution mode, with chopper and time-of-flight (TOF) data acquisition, improved characterization of the scattering features within different *Q* ranges is enabled because of the possibility of varying the wavelength spread Δλ/λ between 2 and 20% (Radulescu *et al.*, 2015 ▸; Amann *et al.*, 2015 ▸). In the extended *Q*-range mode, by means of lenses and a secondary high-resolution detector (pixel size 0.45 mm), a *Q*_min_ down to 1 × 10^−4^ Å^−1^ can be achieved (Radulescu, Szekely *et al.*, 2016 ▸; Radulescu, Pipich, Frielinghaus & Appavou, 2012 ▸) which, in combination with the pinhole mode, permits the exploration of a continuous length scale from 1 nm to 1 µm.

**Figure 1:**
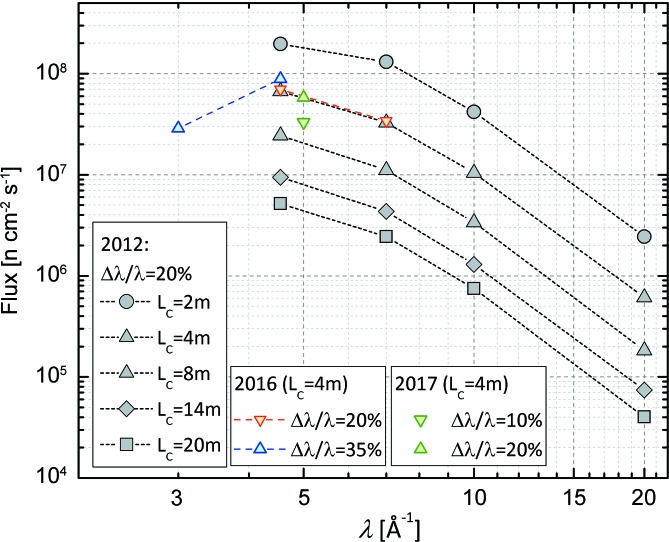
The absolute neutron flux at the sample position on KWS-2 as a function of wavelength λ for different collimation lengths *L*_C_ and full beam size (50 × 50 mm), as provided by the neutron guide. The measurements were carried out using different velocity selectors under different inclination angles with respect to the beam axis.

In the conventional mode, KWS-2 provides a very high neutron flux on the sample (Fig. 1 ▸). This is a result of the combination of a neutron guide system that was specially designed to transport high intensity to the instrument (Radulescu *et al.*, 2012 ▸) and a versatile velocity selector (Astrium GmbH) that enables easy selection between a wavelength spread of 10 and 20%, depending on whether the specific scientific experiment demands either an improved resolution, thus Δλ/λ = 10%, or a high intensity, hence Δλ/λ = 20%. The resolution can be further improved down to Δλ/λ = 2%, in a range where no velocity selector can compete (Radulescu *et al.*, 2015 ▸), by using the TOF mode. The high-flux option enables scientific opportunities in the field of the structural investigation of small soft-matter and biological systems. At high *Q* wavevector transfers these systems typically deliver only very weak scattering signals above the buffer or solvent level. This is the configuration in which the shortest collimation and detection lengths are involved, and it corresponds to the high-flux regime of the instrument. It is very challenging to resolve such weak scattering signals, owing to the requirements regarding the counting rate and stability in time of the pixel response of the detection system. These factors are dictated by the specific conditions of an experiment, especially in the field of biology.

Typically, a SANS study of a biological system deals with the small quantities of sample which are available for investigation, usually of the order of 100 µl, owing to the difficulty in producing special biological samples. Furthermore, a low concentration of the system of interest, *e.g.* protein or biopolymer in solution, is often targeted, because of the interest in characterizing single proteins or polymers. Unfortunately, such samples usually experience instability in solution over short periods, due to aggregation or denaturation tendencies. Finally, the scattering intensities measured from a biological sample must be corrected for several contributions additional to the molecules of interest. These include scattering from the solvent and buffer components in the specific contrast condition and from the sample container. Therefore, constant experimental conditions must be maintained over long periods of time, including all the correction and calibration measurements that are required to obtain the scattering from the investigated system at the end of the experiment.

Some instrumental and data analysis issues that must be taken into account for achieving reliable high-*Q* SANS results may be found in the articles by Barker & Mildner (2015 ▸) and Konarev & Svergun (2015 ▸). On the other hand, practical requirements for applying SANS to biological systems are treated in detail by Gabel (2015 ▸) and Jacques & Trewhella (2010 ▸).

**Figure 2:**
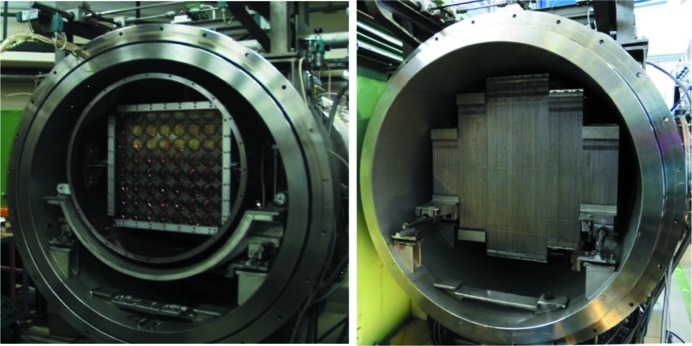
(Left) The old scintillation detector and (right) the new ^3^He detector, during the installation phase on KWS-2.

Until 2015, the main detector on KWS-2 was an Anger camera system based on an array of 4 × 4 ^6^Li glass scintillators and 8 × 8 photomultipliers (Fig. 2 ▸) that provided a 60 × 60 cm active detection area with a space resolution of approximately 7 mm (Kemmerling *et al.*, 2004 ▸). This detection system had a low counting rate at 10% dead time (*ca* 100 kHz) and has shown an inhomogeneous dead time over the somewhat reduced detection area for the case of samples providing strong scattering in the forward direction. Combined with the γ sensitivity, these factors were a drawback with respect to the potential of the instrument in the high-flux regime. Therefore, following collaboration between the JCNS and GE Reuter Stokes Inc., a new high-performance detection system was installed on KWS-2 in the summer of 2015 (Fig. 2 ▸). The new detection system consists of an array of position-sensitive ^3^He tubes with an active detection area equivalent to 0.9 m^2^ and innovative rapid read-out electronics. The detector’s master piece was supplied by GE Reuter Stokes Inc., following the specifications in performance and geometry delivered by the JCNS and the testing of the prototype (Radulescu, Arend *et al.*, 2016 ▸) on KWS-2 in high-flux conditions.

In this paper, we present the features and performance of the new detection system of the KWS-2 SANS diffractometer. We report the implementation of the new detector in the high-flux concept of the instrument, the characterization and adjustment of the detector, and its performance as proven in measurements of some specifically chosen samples representative of the scientific topics developed on the instrument.

## 2. The flux conditions on KWS-2   

**Figure 3:**
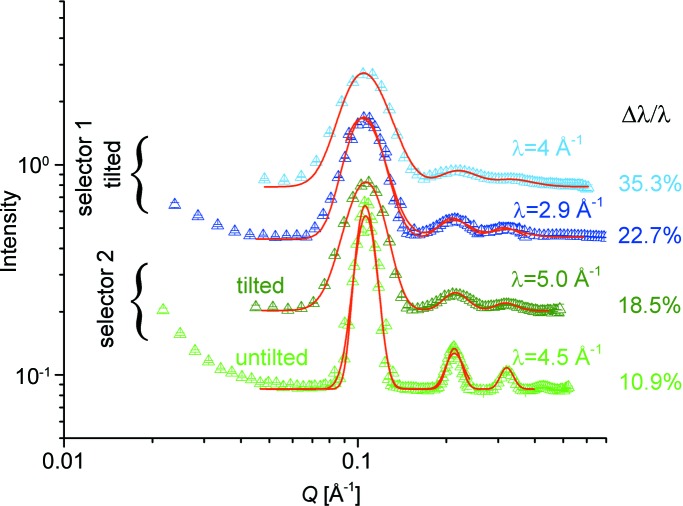
SANS data for the reference AgBeh sample measured on KWS-2 using different selectors positioned at inclination angles of 0° (untilted) and −10° (tilted) with respect to the beam axis. Selector 1 is an Astrium velocity selector with 32 blades (nominal Δλ/λ = 20%), while selector 2 is an Astrium velocity selector with 64 blades (nominal Δλ/λ = 10%). The fits to the data (see text) yielded the Δλ/λ values shown on the right-hand side of the plots.

The neutron intensity distribution and flux along the guide system of KWS-2 and at the sample position were reported in detail in an earlier publication (Radulescu, Pipich & Ioffe, 2012 ▸). For an optimal filling of the cold neutron source (CNS) of the FRM II reactor, the highest flux at the sample position for the shortest collimation length of *L*_C_ = 2 m and λ = 4.5 Å is about 2 × 10^8^ n s^−1^ cm^−2^ (Fig. 1 ▸). The neutron flux is checked periodically by gold foil activation, either at the sample position (monochromatic flux) or in front of the velocity selector (‘white’ flux). KWS-2 is primarily a dedicated high-intensity SANS instrument, which in the instrument concept is provided by a selector that enables a relaxed resolution, Δλ/λ = 20%. Following detailed simulations by *McStas* (Willen­drup *et al.*, 2004 ▸) using a realistic model for the CNS of the FRM II reactor (Zeitelhack *et al.*, 2006 ▸) and the real parameterization of the neutron guide system upstream of KWS-2, it was shown that, by tilting a selector designed for a resolution of Δλ/λ = 10% over an angle of −10° with respect to the beam axis, a gain in intensity based on the resolution relaxation up to Δλ/λ = 20% can be obtained (Figs. S1*a* and S1*b* in the supporting information). The results have been confirmed on the one hand by gold foil measurements in different selector configurations (Fig. 1 ▸), and on the other hand by data from silver behenate powder (AgBeh) collected at different detection distances using different neutron wavelengths λ and different positioning configurations of two selectors, one delivering a standard Δλ/λ = 10% and the other Δλ/λ = 20%. With a similar procedure to that used by Grillo (2008 ▸), the experimental curves collected at one specific wavelength/positioning configuration of the selectors were fitted simultaneously with fixed parameters, either known from small-angle X-ray scattering (SAXS) measurements (peak position and width) or calculated (σ_geo_), Δλ/λ being the fitting parameter for each configuration. The model curves describing the experimental data delivered the wavelength spread Δλ/λ values that are shown in Fig. 3 ▸, which are in good agreement with the simulated results. The fitting procedure was used successfully for the characterization of the KWS-2 chopper system as well (Radulescu *et al.*, 2015 ▸). The use of a velocity selector (Astrium GmbH) that provides either Δλ/λ = 10% or Δλ/λ = 20% (selector 2 in Fig. 3 ▸), depending on whether it is positioned parallel to or inclined with respect to the beam axis, is the main monochromatization method currently used on KWS-2. The setup also enables the possibility of both achieving lower wavelengths down to λ = 3 Å with significant intensity, and continuing to maintain the availability of a very high flux for experiments that require intensity at the expense of resolution by using monochromatic beams with Δλ/λ ≃ 20%.

Hence, for the shortest collimation length, and thus the low-resolution–high-*Q* instrument operation mode, a sample area of 1 cm^2^ can be exposed to more than 10^8^ n s^−1^. Samples that produce strong incoherent scattering, like calibration standards (Plexiglas) and biological samples prepared in H_2_O buffer solutions, usually have a transmission slightly higher than 50% when prepared with a flight path of 1 mm. Ideally, their scattering may generate, on a 1 m^2^ detector placed 2 m after the sample, an intensity which requires a counting rate capability of *ca* 2 MHz for being detected with negligible dead-time losses. However, higher counting rates may occur when larger sample areas or detection distances shorter than 2 m are used. Therefore, the main specified feature of the new detector on KWS-2 is to enable detection of counting rates of at least 2 MHz in routine operation for a dead time of 10%, which is typically the higher accepted dead-time limit in SANS experiments.

## 3. Detector characterization   

As the new detection system is composed of several eight-pack units that work independently of one another (each eight-pack unit has its own processor and electronics), the performance of one detector unit and that of one single tube were tested in advance on KWS-2 in a prototyped eight-pack unit of ^3^He tubes and corresponding electronics. The detailed test aimed to check the fulfilment of the specified parameters for the new detector with respect to the position resolution, the position calibration and linearity, the dead time and counting rate on one eight-pack and one tube, and the stability of the pixel response. The results, reported elsewhere (Radulescu, Arend *et al.*, 2016 ▸), were considered excellent and led to the acquisition of the full detection system from the manufacturer. The tests confirmed a high stability of the pixel response and position, a resolution better than 8 mm and a predicted count rate of several megahertz at 10% dead time for the full detection system. As later tests carried out on different eight-packs confirmed the resolution at the level of the full detector, in the following we will report the characteristics that have a direct impact on the routine use of the detector in high-flux conditions, especially the counting rate and stability.

### 3.1. Detector layout   

**Figure 4:**
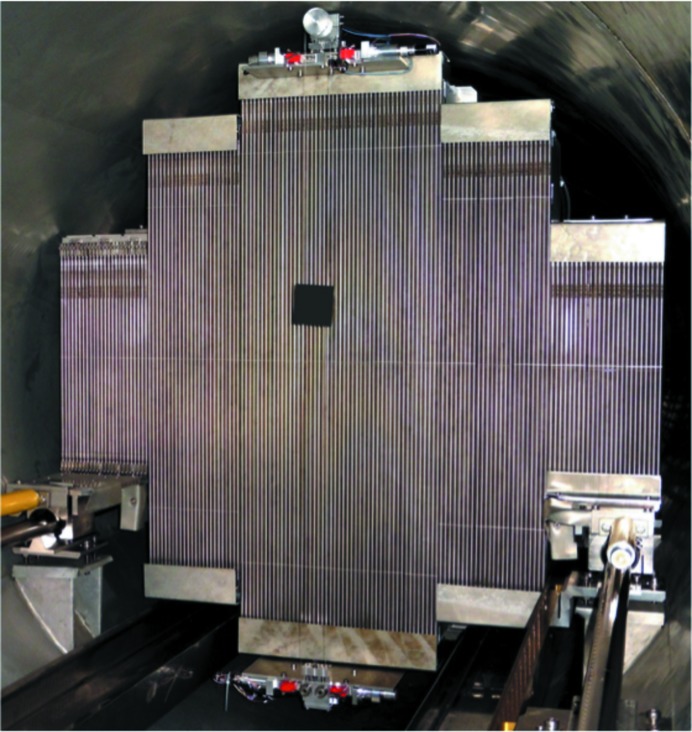
The new detection system on KWS-2 during the installation phase, with the mobile (*x*, *y*) beam stop visible. The beam stop was under adjustment work at the time when the photograph was taken. The upper and lower edges of the tubes were covered in the final state with ceramic borated carbon plates. The beam stop plate is also made of ceramic borated carbon.

The new detector has a modular structure consisting of 18 eight-pack units of ^3^He tubes with a diameter of 8 mm. The eight-packs are combined together in appropriate numbers and lengths, so as to maximize the active area by filling completely the cross section of the KWS-2 tank (Fig. 4 ▸). The high-voltage and readout electronics are mounted in a closed case at the rear of the ^3^He tube panel array (Fig. S2 in the supporting information) and operate at ambient atmosphere under a cooling air stream.

The full system consists of three types of eight-packs with tubes of active length of 372, 822 and 1024 mm. Each eight-pack has two preamplifiers, two high-voltage (HV) modules and a digital processing board (platform processor). The tubes are made of stainless steel (304) and have a wall thickness of 0.25 mm. The ^3^He pressure is 12.6 bar (1 bar = 100 kPa), corresponding to a calculated global efficiency of 85% for λ = 5 Å, which represents the average of the response from the inner cathode edge to the centre of the tube. The calculated total average detection efficiency is about 75%, which includes the geometric gaps in between the tubes and the thickness of the steel tube walls (Mühlbauer *et al.*, 2016 ▸). This value was confirmed by parallel measurements of the same Plexiglas sample in similar conditions with the old scintillation detector (95% efficiency for λ = 5 Å) and the new ^3^He detector. Behind the array of ^3^He tubes, a layer of borated aluminium provides shielding against neutrons that are transmitted through the tube array.

An air box containing two ethernet switches (Hirschmann) and the mechanical (cooling lines) and electrical (wired ethernet cabling for signal and power) interfaces between the eight-packs and the air box and router are attached to the detector panel on the rear. On KWS-2 the detector module operates in a vacuum tank (2 × 10^−2^ mbar). Two air lines (one supply, one exhaust) connect the air box to the outside of the vacuum tank, and cooling air at a flow rate of 1800 l min^−1^ and humidity less than 5% is provided to the air box and further to the electronics of the eight-packs. One optical ethernet cable (for signal) and one power cable are routed through the exhaust air line from the air box to the outside of the vacuum tank and further to another external switch (Hirschman) and power supply (Toellner). The three Hirschmann switches (two in the air box, one outside the vacuum tank) are capable of supporting high-speed data transfer (1 Gbit), power-over-ethernet (PoE) and precision time protocol (PTP).

The working principle of the detection electronics is based on the determination of a neutron event that contains the location and time of the neutron interaction with the detecting gas. The data packets originate in a platform processor that acquires the data, performs the position calculations, time stamps the event in absolute time and sends the data *via* the network connection. The event location is represented by a pixel or ‘detector ID’ and is determined by calculating the ratio between the two ends of the high-resistance anode wire when the charge generated by the ionization is divided into two parts in proportion to the location of the neutron capture. The mapping of the pixel to physical locations is achieved *via* external data acquisition software specific to KWS-2. Accurate time stamping is realized by a ‘time-stamper’ module that consists of a platform processor with special firmware. The time stamper also detects the synchronization pulse from the chopper or other external devices. The HV applied to the detector in operation mode is 1530 V, which represents the factory-determined value for achieving an optimum balance between resolution and intensity performance of the eight-packs. The movement of the detector between defined detection positions after the sample is enabled by the control software of the instrument only after the HV is turned off. When the detection position is reached, the HV is again turned on and data acquisition can commence. Each HV manipulation takes about 30 s.

Finally, the transmitted beam through the sample is caught on a beam stop that consists of a ceramic plate of borated carbon with a size of 70 × 70 × 3 mm. The beam stop is placed at a distance of 10 cm in front of the ^3^He tube array and can be placed at any (*x*, *y*) position within a wide vertical range of *ca* 100 cm and a narrow horizontal range of 6 cm to capture transmitted neutron beams of different wavelength λ for different placements *L*_D_ of the detector after the sample. The beam stop is fixed on one side on a system of nylon threads that are vertically stretched (Fig. 4 ▸). The precise vertical positioning of the beam stop is achieved *via* the encoder of a step motor combined with a triangulation laser sensor (Micro-Epsilon Messtechnik), while the horizontal positioning of the entire system is done by a step motor equipped with an encoder. For measurement of the sample transmission, the beam stop is moved down and an attenuated transmitted beam spot is collected on the detector. The attenuator is placed at the end of the collimation system and can be brought into the beam in a controlled way by the measurement software, synchronized with the downward movement of the beam stop.

### 3.2. Position calibration and geometric corrections   

**Figure 5:**
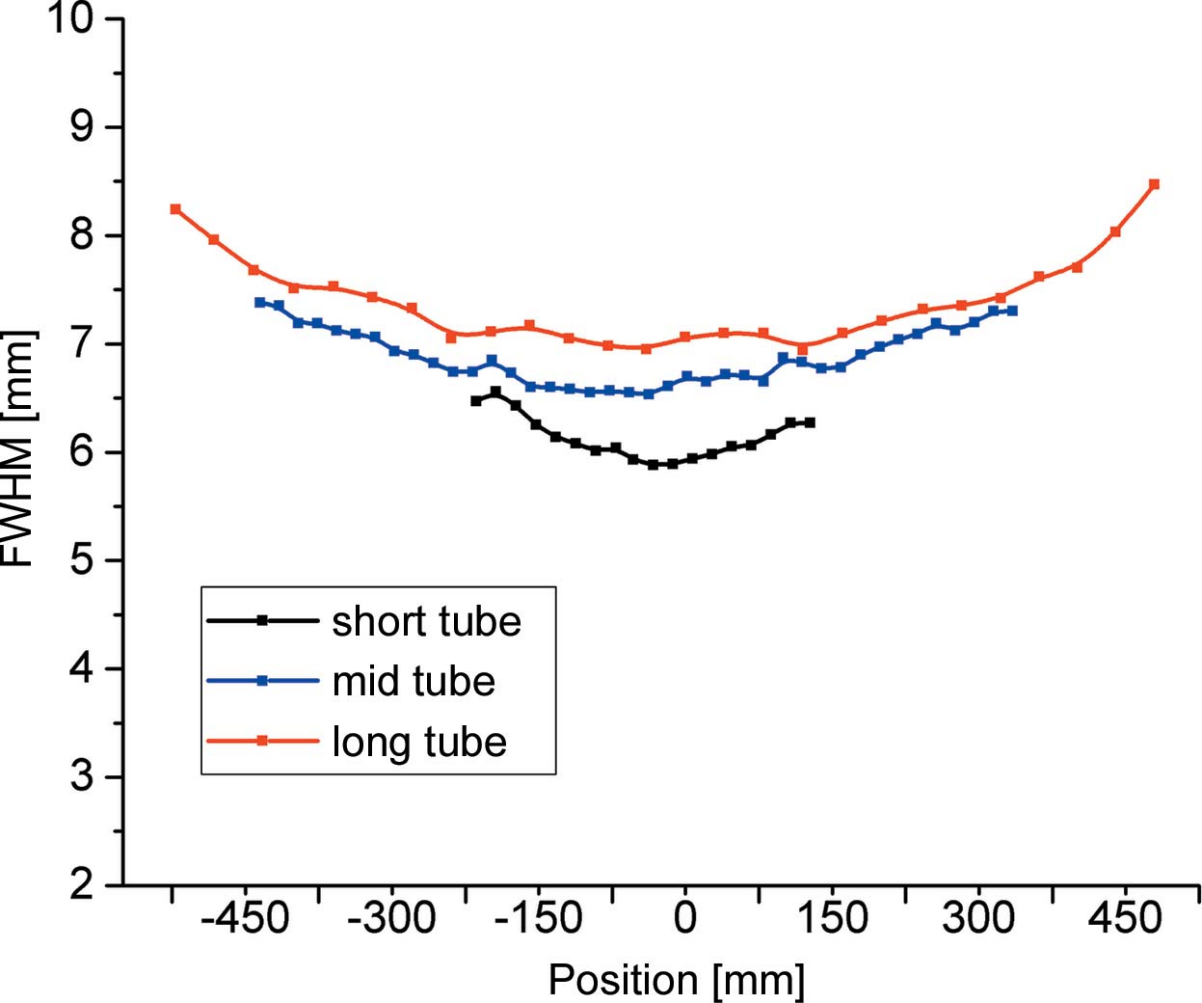
The spatial resolution measured with a pinhole beam along three ^3^He tubes with different lengths that are installed on the KWS-2 detector.

Raw data are collected as a matrix of 144 columns, representing the number of ^3^He tubes, and 1024 lines, representing the number of data reconstruction channels along each tube regardless of its length. The correction for the shift and linear distortion of the active length of each tube (Fig. S3*a* in the supporting information) is achieved by means of a pair of cadmium strips defining a narrow horizontal slit across the detector width, which are mounted right in front of the detector. The horizontal resolution of the two-dimensional detector is determined by the 8 mm pitch of the ^3^He tube array, while the vertical resolution, which was obtained during the prototype test (Radulescu, Arend *et al.*, 2016 ▸) and proven by later tests carried out on every ^3^He tube installed in the detector, is about 6–6.5 mm for the short tubes, about 7–7.5 mm for the mid-length tubes and slightly greater than 8 mm for the long tubes (Fig. 5 ▸). The two-dimensional image with correct positions of events (Fig. S3*b* in the supporting information) is finally converted into a matrix of pixels 8 × 8 mm in size after binning of the 1024 channels along one tube in pixels of 8 mm size each. The effect of the detector resolution on the total *Q* distribution 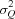 is very small and does not affect the quality of the data measured in conventional pinhole mode (Fig. S4 in the supporting information).

To take advantage of the high intensity available on KWS-2, and the high volume efficiency of each tube, as estimated by the producer, we designed a planar arrangement of the ^3^He tubes that offers a convenient way of correcting the data for geometric effects, including shadowing effects and the tube geometry (He *et al.*, 2015 ▸). A planar arrangement of ^3^He tubes is used in the detectors in operation on D22 (Dewhurst, 2008 ▸) at ILL, Grenoble, France, V4 (Keiderling & Wiedenmann, 1995 ▸) at HZB, Berlin, Germany, and SANS-1 (Mühlbauer *et al.*, 2016 ▸) at FRM II, Garching, Germany. Zigzag arrangements were also employed in the detectors on some SANS instruments, such as GP-SANS and Bio-SANS (He *et al.*, 2015 ▸) or SANS-J-II (Noda *et al.*, 2016 ▸), Tokai, Japan. However, a zigzag ^3^He tube arrangement requires a much more complicated correction procedure, as well as much higher costs for detector production.

### 3.3. Counting rate and dead time   

The counting rate capability of a SANS detector is typically measured using an incoherent scattering sample at a short detection distance, leading to a high but rather homogeneous distribution of intensity on the detector. The measured counting rate *N*_M_ should be compared with the expected scaled counting rate (true counting rate *N*_T_), as measured in an identical setup with a dead-time-free monitor (fission chamber). Either the sample slit size (beam size) or the number of identical attenuators can be varied, which will yield a variation in the counting rate as a function of beam size or attenuator thickness. This variation can also be interpreted in a straightforward way. At low values the counting rate behaviour is linear, while for high values deviations like *N*_M_ < *N*_T_ are expected as a result of dead-time effects. Several models can describe the counting rate behaviour (Knoll, 1979 ▸), which will deliver the dead time due to ‘inactivity’ of the detector during the time needed to collect the charges and the electrons (the intrinsic dead time of the tubes themselves) and produce and transfer the data (limitations of the read-out electronics). Detailed presentations of the procedures for determining the maximum counting rate and the dead time at SANS instruments are given by Grillo (2008 ▸) and Strunz *et al.* (2004 ▸). This correction is needed to provide the true scattered intensity from the sample and enable a reliable quantitative analysis of the scattering curves expressed in absolute units after calibration against a standard sample with known scattering cross section (vanadium, water or Plexiglas). Typically, in a SANS experiment several detection–collimation conditions are used. With proper dead-time corrections, the curves collected at different setups must overlap and merge together in a final scattering curve that covers a broad *Q* range.

For the detector prototype, the measured count rate *N*_M_ on the eight-pack was interpreted by the paralysable model, which delivered a 10% loss at around *N*_M_ = 700 kHz (Radulescu, Arend *et al.*, 2016 ▸). Given that each eight-pack module has its own platform processor, it was expected that, in an ideal case, the full two-dimensional detector would provide a count rate that would scale with the number of eight-packs.

**Figure 6:**
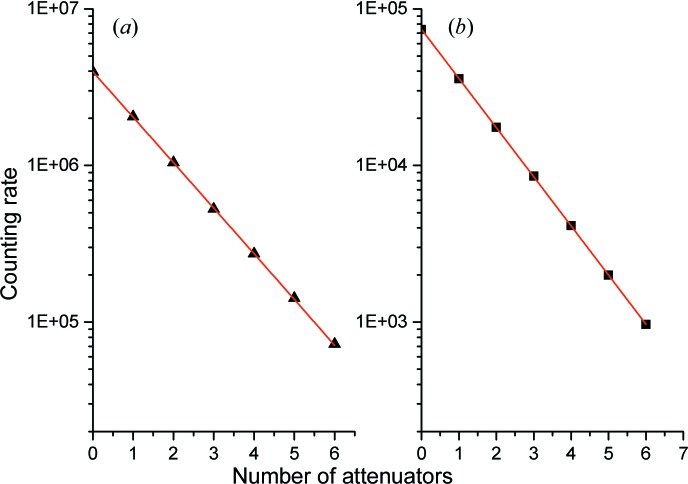
(*a*) Integral (whole area) and (*b*) localized (spot on one tube) count rates from a Plexiglas sample, measured using a variable number of borosilicate glass plates of similar transmission as attenuators of the incoming beam. The variation in count rate with attenuator thickness is linear on the logarithmic scale.

The counting rate and dead time of the entire detector system was controlled in two ways for homogeneous coverage (flat pattern) by using the incoherent scattering from a Plexi­glas sample. The first method involved varying the number of borosilicate glass attenuators with identical thickness and transmission placed in front of the Plexiglas sample and after the sample slit, keeping the instrument setup unchanged. The second method involved varying the collimation entrance slit by keeping the sample slit constant and hitting the Plexiglas sample with a full non-attenuated beam. In both cases, the detector was placed 1.1 m after the Plexiglas sample and the transmitted direct beam was captured by a beam stop placed in front of the detector. Additionally, using a narrow beam and parking the beam stop on the bottom of the detector, the dead time of one tube was determined for a localized intensity by varying the incoming flux in a gradual way by inserting a variable number of attenuators after the sample aperture. This is meant to check the dead time for the case of transmission measurements, when the attenuated direct beam will be caught on the detector as an intense localized spot. Figs. 6 ▸(*a*) and 6 ▸(*b*) show the counting rate evolution collected using the procedure involving attenuators to determine the flat pattern and localized intensity dead times. For both methods described here, no deviation from linear behaviour is observable up to the maximum counting rates reached in both configurations.

**Figure 7:**
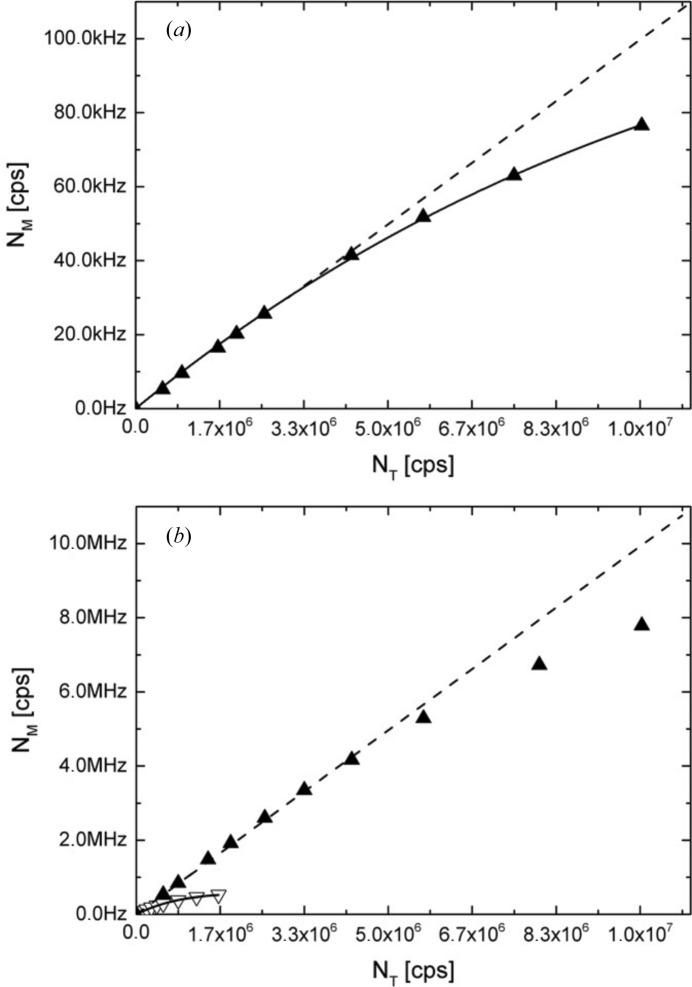
The counting rate capability of the new detection system. (*a*) The dead time per tube extracted from the fit of the measured counting rate *N*_M_ (filled triangles) with the paralysable model (solid line) and (*b*) the overall counting rate. The measured and fitted results are presented as a function of the sample aperture size converted into theoretical count rate *N*_T_. The dotted line represents the ideal behaviour of the count rate in the absence of dead time. The open symbols in panel (*b*) represent the count-rate variation on the old detector, which was also fitted with the paralysable model (solid line).

Fig. 7 ▸(*a*) presents the response of one long tube obtained by varying the beam size on the Plexiglas sample and the fit of the counting rate behaviour with the paralysable model (Knoll, 1979 ▸). Dead-time effects can be observed at counting rates of 35 kHz and higher, with a dead-time constant of 3.3 µs that corresponds to a 10% loss at around 50 kHz. The capability of the new detector is revealed by the overall counting rate of the detector (144 tubes), which is presented in Fig. 7 ▸(*b*) together with the counting rate behaviour of the old scintillation detector. Dead-time effects can be observed at counting rates of 5 MHz and higher, with a 10% loss at around 6 MHz.

Although not matched, the results obtained for the full detection system are very close to the prediction made by the ideal extrapolation of the counting capability of a single eight-pack during the prototype test.

This indicates that the full system has some limitations due to the time needed to collect the charges (the intrinsic dead time of the tubes themselves), and to amplify and digitize them (limitations of the read-out electronics). The position calculations are performed at very high speed on the platform processor and thus do not contribute to the dead time of the detector. Another limitation may be imposed by the transfer of data to the acquisition computer.

Nevertheless, for routine operations on KWS-2, counting rates of about 0.5–1 MHz are realistically practical.

### 3.4. Stability of the pixel response   

Stability of the pixel response over a long time is a key property of a SANS detection system. Typically, an experimental SANS session extends over a couple of days, including not only the measurement of the samples of interest and their references (solvents or buffer solutions), but also any additional correction and calibration measurements, such as those of the empty can, blocked beam, standard sample *etc*. The intensity collected from the sample of interest *I*_S_ and from the standard sample *I*_St_ can be expressed as follows:
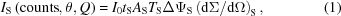


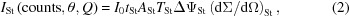
where *I*_0_ represents the incoming intensity (delivered by the collimation system), *t* is the thickness, *A* is the area exposed to the beam, *T* is the transmission and ΔΨ is the solid angle in which a detection cell is seen from the sample position. If both the sample and the standard are measured under the same conditions with respect to the incoming beam (*L*_C_, *A*_C_, *A*_S_, λ and Δλ/λ), and thus the same *I*_0_ and *A*, and if the solid angle is expressed as *A*_D_/(*L*_D_)^2^ (with *A*_C_ the area of the collimation aperture and *A*_D_ the area of a detection cell), then by dividing the two relations the scattering cross section of the sample is obtained as
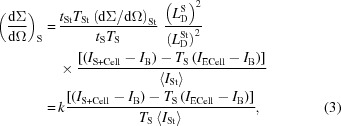
where *I*_St_ is expressed as an average 〈*I*_St_〉 (the standard, since an incoherent scattering system delivers a flat scattering pattern), and *I*_S_ is obtained after correcting the measured intensity of the sample in the cell (container) for the contribution of the empty cell *I*_ECell_ and the background on the detector for the blocked beam, *I*_B_. The calibration factor *k* is calculated by the data-reduction software, based on the scattering from the standard sample and the geometric parameters of the sample (basically, the thickness *t*_S_). The factor μ = *t*_St_ *T*_St_(dΣ/dΩ)_St_, which contains the scattering and physical parameters of the standard sample, depends on the neutron wavelength λ and is typically known from the calibration of the standard sample. Biological samples such as low concentrations of proteins in buffer solutions require a long acquisition time, of the order of several hours, depending on the flux conditions (*L*_C_, *A*_C_, *A*_S_, λ and Δλ/λ) and the detection distance *L*_D_. To achieve the scattering cross section of the macromolecule under investigation in a reliable way, the terms *I*_S+Cell_, *I*_ECell_, *I*_St_ and *I*_B_ in equation (3), where S is either protein + buffer or buffer, must be measured under certain stability and background conditions on the detection system. These measurements require stability over longer timescales, in order to include the additional measurements involved in the correction procedure. This is in addition to the requirement for stability at different high counting rates, which are typically experienced during measurements performed in the high-*Q* regime, suitable for the investigation of small mol­ecules.

**Figure 8:**
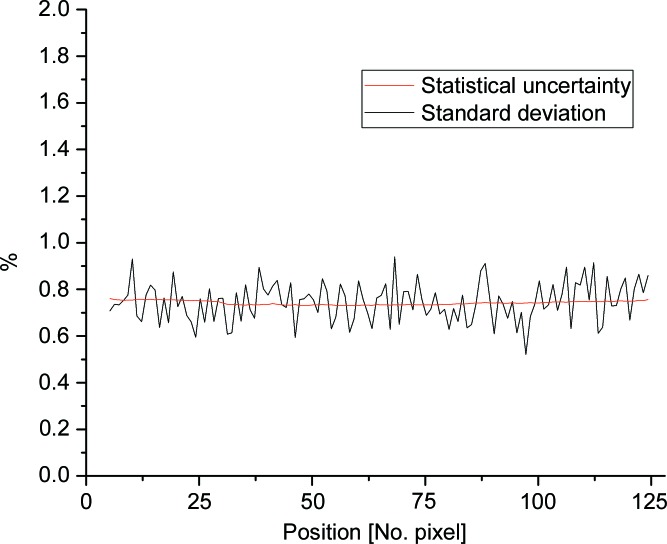
The standard deviation and statistical uncertainty calculated for each pixel of a long ^3^He tube based on 20 similar measurements, 0.5 h each, carried out on a Plexiglas sample with an overall count rate of 125 kHz.

The intrinsic electronic background of the detection system is about 1 count s^−1^ over the entire detector. The stable operation of the read-out electronics is favoured by a constant temperature on the boards and field-programmable gate array circuits in the working regime (Fig. S5 in the supporting information). As mentioned in §3.1, the detection system is isolated electro­magnetically, and therefore electromagnetic interference disturbances are minimized. These conditions enable a high stability of the pixel response. This is periodically checked through long series of similar Plexiglas measurements carried out at a constant counting rate during the day and the night, when the electromagnetic compatibility and background conditions around the instrument are different. Fig. 8 ▸ shows the statistical deviation and statistical uncertainty of the intensity on each pixel of a long detector tube based on 20 similar runs on Plexiglas, each 0.5 h long, at an overall counting rate of 125 kHz. The measurements yielded a mean standard deviation of 0.742%, which is consistent with the mean statistical uncertainty of 0.744%. These results prove that the pixel rate stability lies within statistical uncertainty, a feature that was also observed during the test of the prototype for higher counting rates.

**Figure 9:**
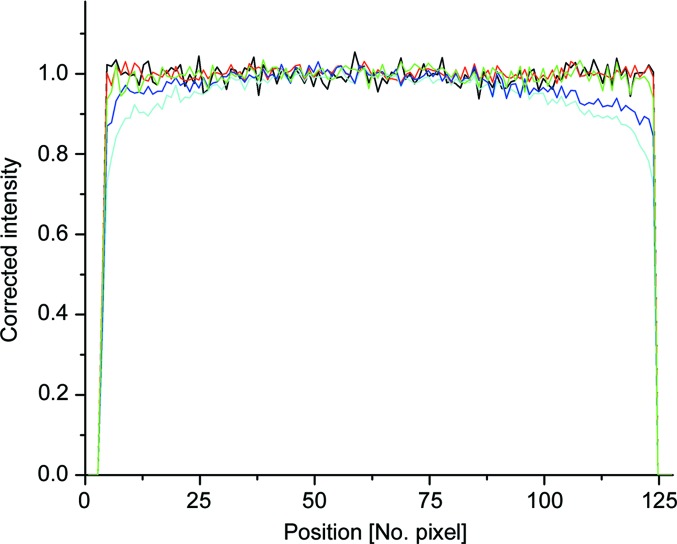
The intensity profile for one long ^3^He tube collected at different count rates and corrected for the detector sensitivity measured at different count rates. The curves were obtained as follows: red denotes measurement at 2 MHz corrected with sensitivity at 2 MHz; black denotes measurement at 500 kHz corrected with sensitivity at 800 kHz; green denotes measurement at 2 MHz corrected with sensitivity at 800 kHz; blue denotes measurement at 3.2 MHz corrected with sensitivity at 2 MHz; and cyan denotes measurement at 5 MHz corrected with sensitivity at 2 MHz.

**Figure 10:**
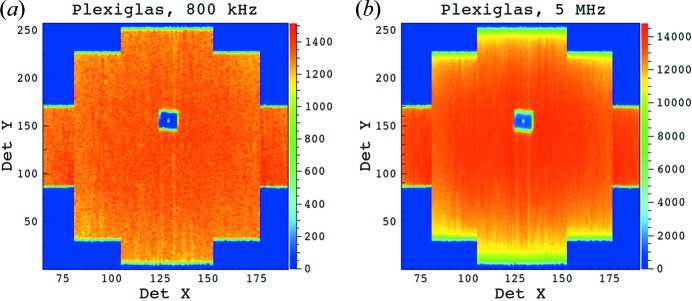
The two-dimensional raw data patterns from the Plexiglas sample, collected at two high count rates on the new detector placed at *L*_D_ = 1.1 m. The data measured at the 5 MHz count rate show a vertical distribution of the intensity with clear drops towards the ends of the long and mid tubes, while the short tubes still show a homogeneously distributed intensity.

**Figure 11:**
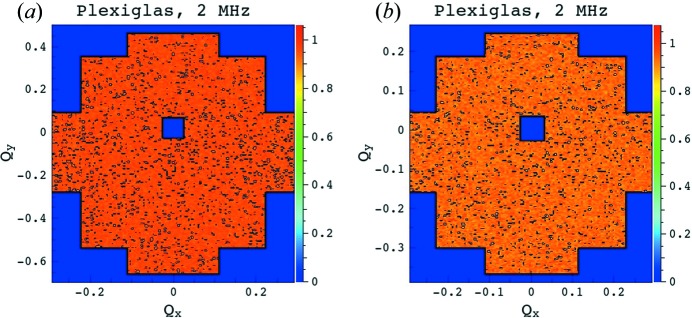
The two-dimensional sensitivity-corrected patterns from the Plexiglas sample, collected at a count rate of 2 MHz on the new detector placed at *L*_D_ = 1.1 m. The sensitivity corrections were achieved with sensitivity measurements at count rates of (*a*) 1.8 MHz and (*b*) 800 kHz.

The ratio between data collected on a long ^3^He tube for similar and for different counting rates (Fig. 9 ▸) stays constant along the entire tube length (end pixels were excluded owing to the high uncertainty) for counting rates up to *ca* 2.5 MHz. Figs. 10 ▸(*a*) and 10 ▸(*b*) show the two-dimensional raw data as they were collected at counting rates of 800 kHz and 5 MHz, respectively, while Figs. 11 ▸(*a*) and 11 ▸(*b*) show the data collected at a counting rate of 2 MHz and corrected with detector sensitivities measured at 1.8 MHz and 800 kHz, respectively. The results confirm a good data correction in both cases.

The detector sensitivity corrections reported in Fig. 9 ▸ reveal that, for very high counting rates above 3 MHz, deficiencies in the position reconstruction appear, which lead to small shifts in the positions towards the centres of the tubes. The same effect was observed for the mid-length tubes. Consequently, for such very high counting rates, corrections must be performed which take into account measurements of in­coherent scatterers (flat pattern) at similar counting rates. Therefore, the counting rate limit for the new detector to generate data in routine operation, which can be straight­forwardly corrected, should be considered as 3 MHz.

## 4. Detector performance   

During the performance characterization of the new KWS-2 detection system with ^3^He tubes and rapid read-out electron­ics, several features were highlighted: the calibration of the standard samples used on KWS-2; the advantage afforded by the larger active area to improve the maximum possible wavevector value *Q*_max_; the instrumental resolution and possibility of resolving in standard mode scattering features from objects with a low size polydispersity; the possibility of using event-mode data acquisition in the investigation of fast structural transformations with a time resolution at the level of 1 ms; and the benefits of the available high counting rate for the characterization of small proteins at concentrations as low as 1 mg ml^−1^ or even 0.5 mg ml^−1^ in buffer solution.

### 4.1. Wavelength and intensity calibration   

Wavelength calibration is carried out using the Bragg reflections from silver behenate (AgBeh powder, supplied by Chemos GmbH, Germany), which is one of the very few materials that feature cold neutron Bragg reflections in the SANS angular range (Gilles, *et al.*, 2000 ▸). These reflections (Fig. 3 ▸) can be analysed with Bragg’s law to determine the wavelength of the primary beam delivered by the velocity selector (Grillo, 2008 ▸), knowing the long-period spacing *d* = 58.380 Å of AgBeh. The large particle size of the AgBeh powder yields the scattering observed at low *Q*. The three Bragg peaks which were observed using different *L*_D_ values within the range 1.5–4 m (as determined using a Theis TCL 8000 cross-line laser with an accuracy of ±1 mm over a range of 5 m) and various velocity selector speeds were analysed, and the absolute wavelengths were determined for different positioning orientations of the velocity selector with respect to the beam axis.

**Figure 12:**
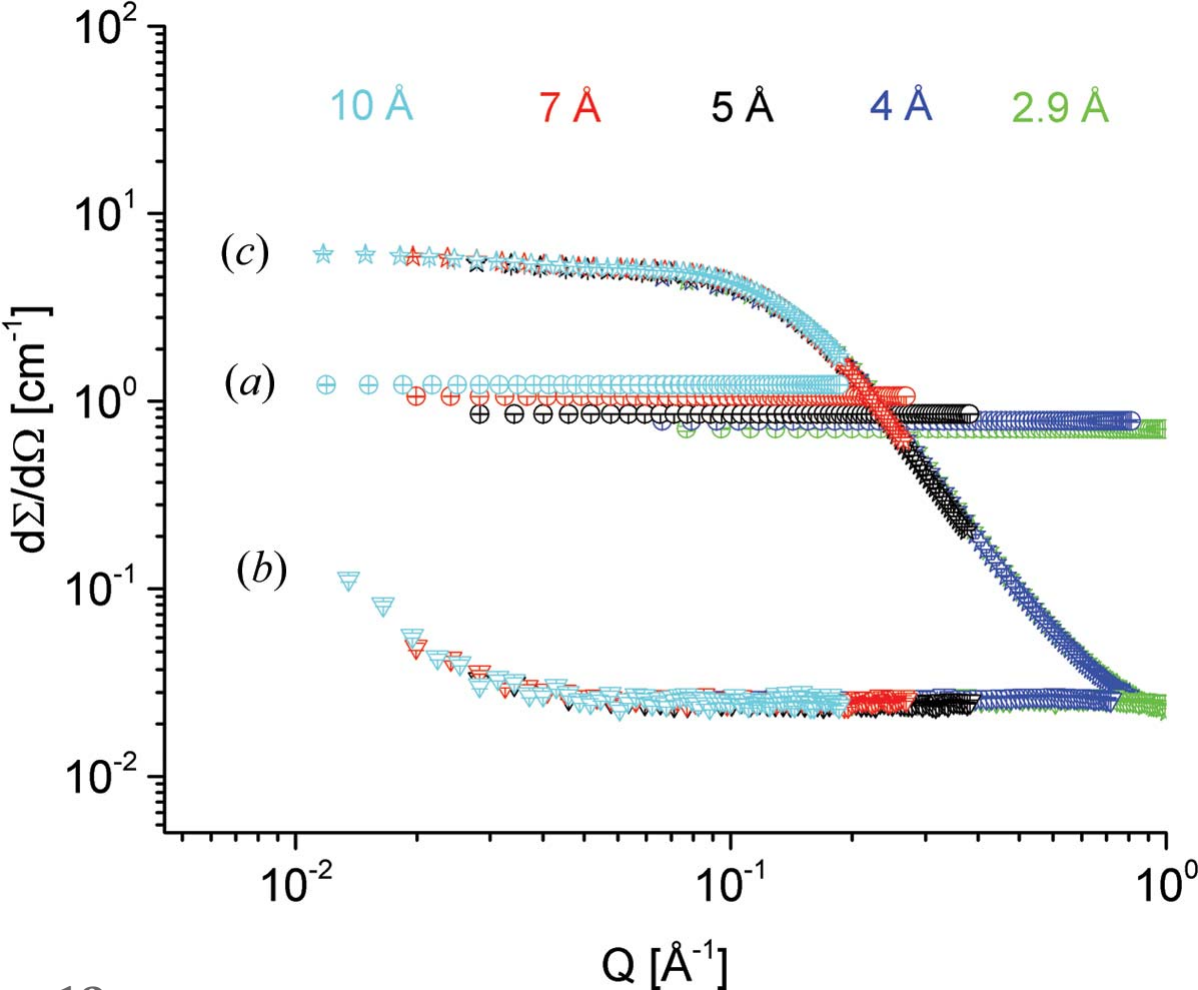
Absolute scattering cross sections from (*a*) the Plexiglas secondary standard, (*b*) vanadium and (*c*) glassy carbon for different wavelengths. The data were calibrated with vanadium and cross-checked with glassy carbon.

The scattering cross section dΣ/dΩ of the system of interest is obtained in absolute units at the end of the SANS experiment by calibrating the measured intensity from the system against the known scattering properties of a standard sample. In neutron scattering experiments vanadium is typically used as a standard sample. However, because of the weak scattering of vanadium, vanadium-calibrated materials with stable physical properties are typically used as secondary standards for routine SANS experiments. The secondary standard sample used on KWS-2 is a Plexiglas slab with a thickness of 1.5 mm. The μ factor in equation (3) characterizing the scattering properties of the Plexiglas is periodically controlled in parallel measurements with vanadium (Fig. 12 ▸) carried out with different neutron wavelengths and cross-checked with glassy carbon (Zhang *et al.*, 2010 ▸), a standard sample that scatters completely coherently and is typically used in SAXS. The results from glassy carbon are well superimposed over each other regardless of the wavelength. In contrast, the data from Plexiglas show deviations from each other due to multiple scattering and inelastic scattering effects, the intensity level increasing with increasing wavelength. The multiple scattering and inelastic scattering effects in SANS on hydrogenated materials are treated extensively by Barker & Mildner (2015 ▸). The use of Plexiglas as the intensity standard on KWS-2 has a historical reason related to the limitations imposed by the old detection system. For this system, identical optical conditions were selected for the measurement of sample and standard, so that the Plexiglas delivered flat scattering patterns at counting rates which the old detector could operate at with a dead time lower than 10%. On the other hand, under similar conditions coherent scattering standards, like glassy carbon, would provide much higher counting rates, close to 1 MHz, which were beyond the capabilities of the old detector. Such counting rates can now be appropriately detected with the new detection system, which makes glassy carbon an attractive intensity standard alternative to Plexiglas.

### 4.2. Two-dimensional scattering patterns and *Q*_max_   

**Figure 13:**
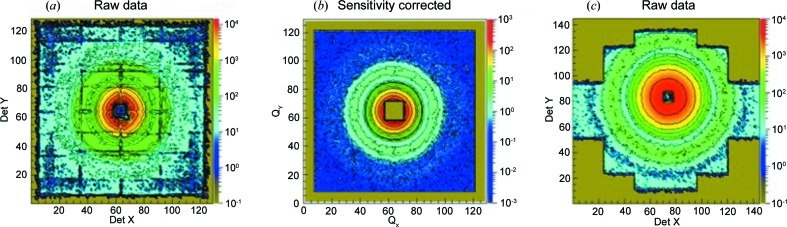
Typical scattering patterns collected with the old scintillation detector as (*a*) raw data and (*b*) data corrected for detector sensitivity, and (*c*) with the new ^3^He detector as raw data.

Fig. 13(*a*) ▸ shows a typical two-dimensional raw scattering pattern as observed from the old scintillation detector. The grid-like structure corresponds to the detector regions with lower sensitivity, due to the limited size of the scintillation plates glued together in a 4 × 4 array and the 8 × 8 photomultipliers. Detector sensitivity corrections had to be carefully applied to the raw data (Fig. 13 ▸ *b*), which sometimes made live analysis during an experiment difficult. The typical two-dimensional scattering pattern collected with the new ^3^He detector is presented in Fig. 13 ▸(*c*). The installation of the larger active area in the predefined configuration of the detector tank leads to an off-centre aspect of the scattering patterns. The new detector cannot be moved vertically or horizontally inside the tank. It is only because of the mobility of the beam stop that masking of the direct beam in different positions on the detector can be achieved. For typical counting rates used in routine measurements, the raw data and sensitivity-corrected data on the new detector look the same.

**Figure 14:**
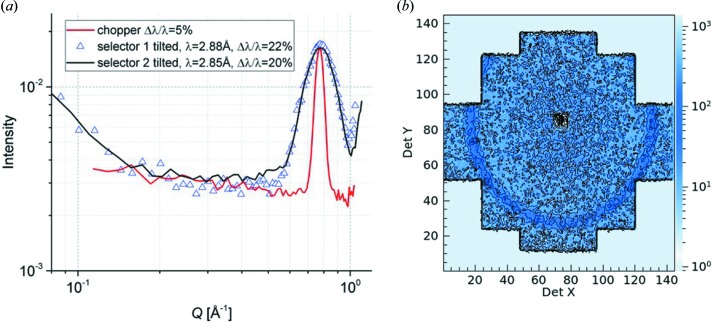
(*a*) One-dimensional scattering patterns from fullerene, C_60_, measured in different configurations on KWS-2. (*b*) The two-dimensional scattering pattern, showing the observation of the ring yielded by the (111) crystalline peak of C_60_ on the active area of the new detector.

Combining the large detection area of the new detector with the use of neutrons with λ = 3 Å delivered by the tilted selector enables the highest value of the wavevector transfer *Q*_max_ to be pushed towards *Q* = 1 Å^−1^. Fig. 14 ▸ presents the scattering pattern from C_60_ fullerene (Sigma–Aldrich GmbH) collected in the high-*Q* region on KWS-2 with different wavelength resolutions. The (111) crystalline peak of C_60_ is clearly revealed at *Q* = 0.77 Å^−1^, in agreement with the peak position observed in other studies (Sanz *et al.*, 2015 ▸). These measurements demonstrate that *Q*_max_ = 1 Å^−1^ can be reached on KWS-2 and that the wavelength resolution Δλ/λ = 20% regardless of which of the two selectors on KWS-2 was used, owing to the wavelength cut-off of the neutron guide (Fig. S1 in the supporting information).

### 4.3. Size standard samples   

**Figure 15:**
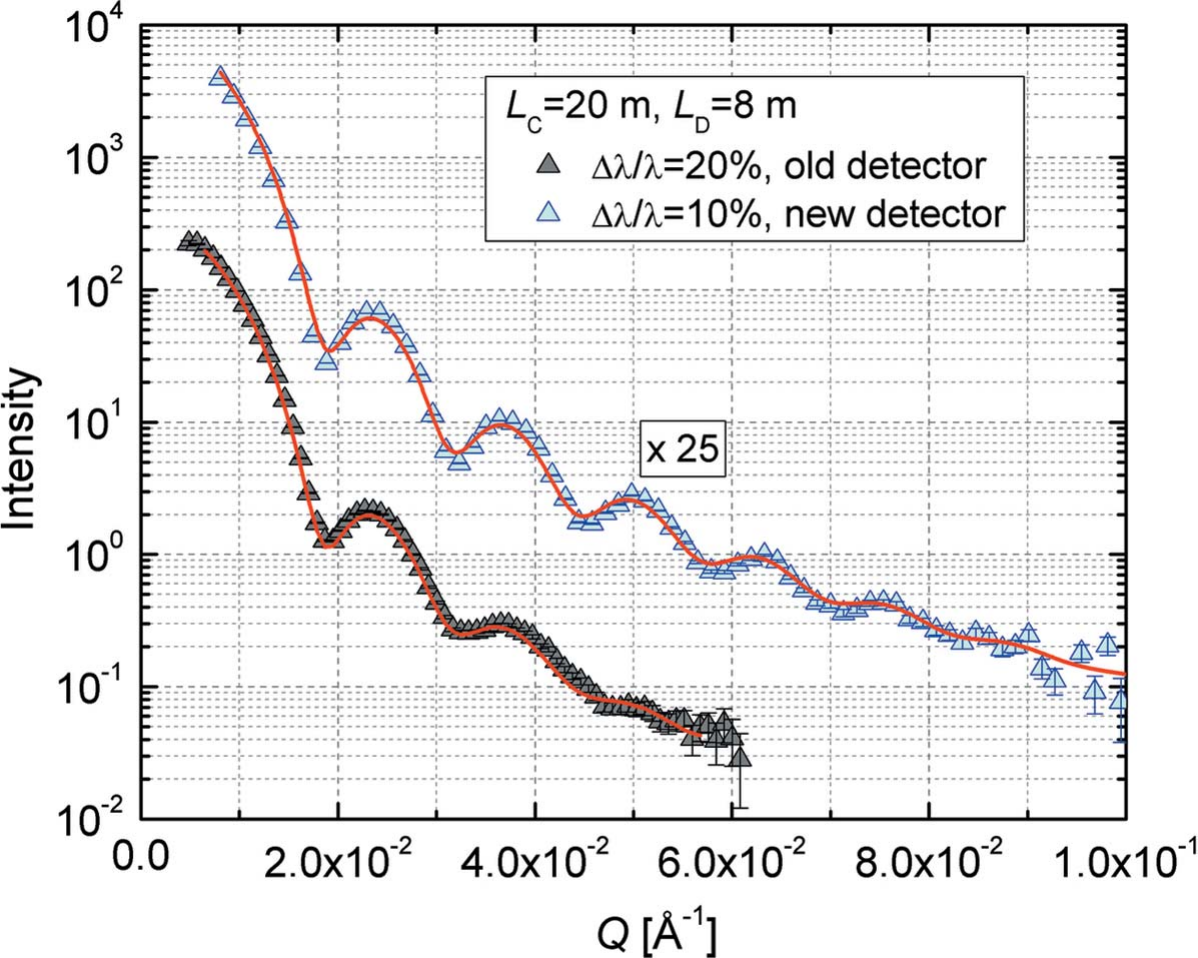
SANS curves of SiO_2_ particles in d-DMF, both measured at a detector distance of 8 m with the old (black triangles) and new (blue triangles) detectors on KWS-2. The fits correspond to equation (9) of Radulescu *et al.* (2015 ▸).

SiO_2_ particles of a known size (*R*_0_ = 241.3 Å) and narrow size distribution (σ_0_ = 0.0323) have been used in the past to check the resolution of the instrument (Radulescu *et al.*, 2015 ▸; Vad *et al.*, 2010 ▸). Fig. 15 ▸ shows the scattering cross section from such particles in deuterated dimethylformamide (d-DMF) solution measured at the same detection distance with the old and new detectors on KWS-2. The wavelength and collimation length were the same, although the wavelength spread was different. For the measurement carried out with the new detection system, Δλ/λ = 10% was provided by the new velocity selector used in a position parallel to the beam axis. The particle volume fraction in solution did not have any structure-factor effect on the scattering curves. The fit to the experimental data, which took into account the experimental resolution and the polydispersity effect, described the experimental curves very well and delivered the correct size for the particles. The plot clearly reveals the advantage of the new detector, with its larger active area, over the old detector.

### 4.4. Event-mode option   

A neutron event is the fundamental measurement of the neutron detection system by which the location and time of the detector–neutron interaction are determined.

The new detector enables data collection in event mode, which represents an advantage for time-resolved SANS (TR-SANS) experiments aimed at investigating fast changes in sample morphology with sub-millisecond time resolution. Such changes may occur when specific fields are applied to a sample, *e.g.* oscillatory magnetic or electric fields, or steady-state or oscillatory shearing. In a time-resolved SANS experiment carried out in a standard configuration, the best time resolution that can be reached is defined by the wavelength λ, the wavelength spread Δλ/λ provided by the velocity selector and the detection distance *L*_D_. Thus, for a sample-to-detector distance *L*_D_ = 8 m and a wavelength λ = 5 Å with Δλ/λ = 10%, the best time resolution that can be achieved is 2 ms. This will worsen in the case of a longer detection distance or a larger wavelength spread. To improve the time resolution a chopper must be used in addition to the velocity selector. Using the chopper on KWS-2 (Radulescu *et al.*, 2015 ▸) a pulsed beam of a certain frequency and pulse width is produced, so that an improved time resolution, as good as 0.5 ms, can be achieved on the detector for experiments at a long detection distance *L*_D_. The improvement in the time resolution is of course accompanied by a loss in intensity (Radulescu *et al.*, 2015 ▸). Nevertheless, using event-mode data acquisition no synchronization of the pulsed field on the sample and the pulsed beam is necessary, as is required in the TISANE method (Kipping *et al.*, 2008 ▸). In event mode, each neutron is detected at an absolute time and position on the detector. In this case, integration of the neutron arrival events to form complete SANS data sets is performed after data acquisition, using synchronization between the time stamp of each neutron and the time behaviour of the field applied on the sample, which can be achieved even though each of the two time coordinates is recorded separately.

As a proof of principle of event-mode data acquisition using the chopper to improve the time resolution on KWS-2, we carried out a rheology TR-SANS (rheo-TR-SANS) experiment on a solution of F127 Pluronic polymer in D_2_O at a polymer volume fraction of Φ_pol_ = 5%, using an Anton Paar MCR-500 rheometer. The ordering of the F127 Pluronic polymer formed in different solvents, which is directed by large-amplitude oscillatory shearing of the micellar phase, has been intensively studied and reported in detail in previous papers (Hyun *et al.*, 2006 ▸; López-Barrón *et al.*, 2012 ▸). Our aim is not to report another rheological study on this well known system, but rather to present the improvement gained in the experimental conditions on KWS-2, specifically that enabling such studies with shorter time resolutions than can be provided by the standard measurement mode, in combination with a sufficiently high intensity which can now be achieved by recording all neutrons delivered by the tandem velocity selector, *i.e.* the chopper.

**Figure 16:**
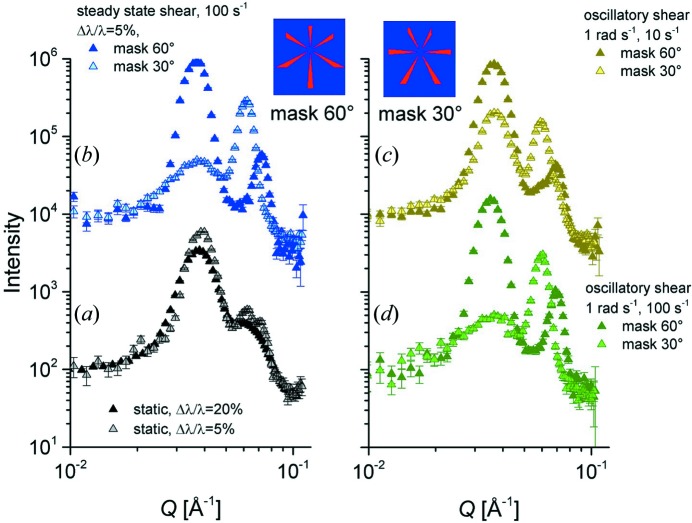
One-dimensional scattering patterns from the Pluronic 127 system in D_2_O (Φ_pol_ = 5%), (*a*) in the initial relaxed state and (*b*) under steady or (*c*), (*d*) oscillatory shearing performed with a rheometer in couette geometry. The data were measured with different setups (Δλ/λ = 20% or Δλ/λ = 5%) and averaged either radially (in the relaxed state) or over two angular sectors (under shearing), as indicated by the two masks shown in the upper part of the figure.

The experiments were carried out using a detection distance *L*_D_ = 8 m. Pluronic F127 forms a correlated micellar system in D_2_O, which in quiescent conditions generates rings in the SANS pattern. Radial averaging produces a series of peaks, which can be better resolved when a better resolution in Δλ/λ is involved (Fig. 16 ▸ *a*). Applying steady-state shearing causes the micelles to order in a hexagonal close-packed (h.c.p.) layered structure, generating ordered spots in the two-dimensional scattering pattern. The scattering pattern can be analysed under different angular sectors in order to resolve the structure revealed by the first- and higher-order correlation peaks (Fig. 16 ▸ *b*). Shear ordering and melting and recrystallization of the micellar arrangement in a cyclic mode can be investigated by applying large-amplitude oscillatory shear (LAOS), as reported by López-Barón *et al.* (2012 ▸).

In our test experiment, the rheometer used in couette geometry enabled the application of either steady-state or oscillatory shearing on the sample, by varying the shear rate and the frequency and strain amplitude conditions. In oscillatory rheo-TR-SANS (ω = 1 rad s^−1^), every neutron was collected with its assigned time, and the measured intensities, either global or integrated on a certain correlation peak, were processed after acquisition in a manner synchronized with the oscillatory cyclic state of the sample. A similar procedure was used by Adlmann *et al.* (2015 ▸) in neutron reflectivity measurements. The data were collected over a total acquisition time of 1 h for different strain rates (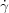 = 10 and 100 s^−1^) and then integrated in a different number of time slots during a cycle of the rheometer, depending on the targeted time resolution.

**Figure 17:**
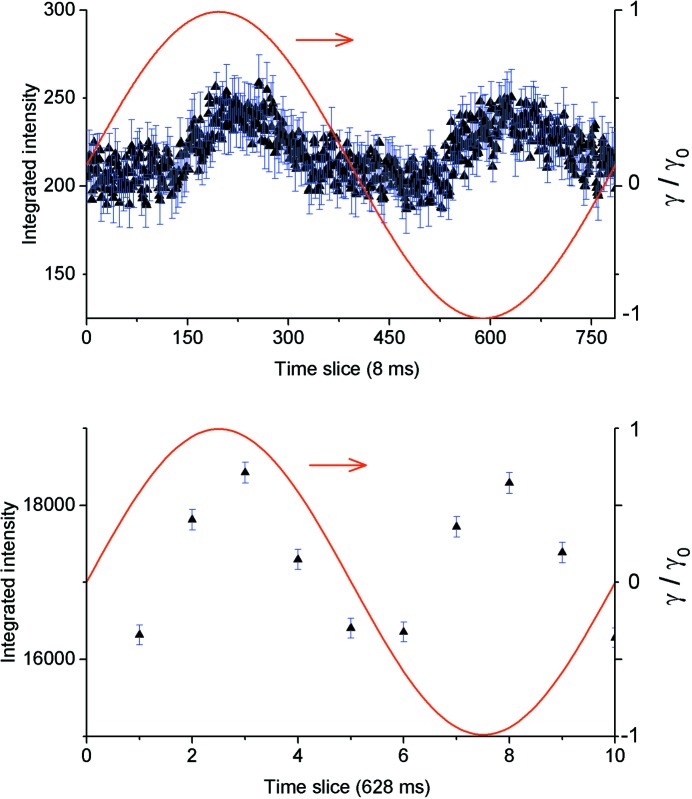
The variation in integrated intensity over a single first-order diffraction spot as a function of time during a rheometer cycle, binned in a different number of time slices according to the intended time resolution. The red curves represent the normalized strain amplitude.

For the oscillatory shearing measurements, the variation in integrated intensity over the first-order diffraction spots within a rheometer cycle is indicative of the evolution of the sample structure as a function of the periodic excitation. In our data interpretation we used the same approach as López-Barrón *et al.* (2012 ▸). Fig. 17 ▸ presents the integrated intensity corrected for the isotropic halo for ω = 1 rad s^−1^ and 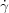 = 10 s^−1^, binned within one rheometer cycle in a different number of time slices. Two channel widths were considered, which correspond to two extreme approaches, namely 628 and 8 ms. Over a total acquisition time of 1  h, the integrated counting times in each channel in the two situations considered here were 360 s and about 4.6 s, respectively. The effect of the decreased statistics in relation to the increased time resolution is visible in the difference in the lengths of the error bars. The intensity varies during the oscillatory excitation. Within a cycle, the maximum of the corrected spot intensity is observed after reaching the maximum strain amplitude γ (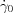 = γ_0_ω), which is in agreement with the observations reported by López-Barrón *et al.* (2012 ▸). The angular analyses of the two-dimensional scattering patterns obtained from different strain-rate conditions in the position of the maximum strain amplitude (Fig. 17 ▸, bottom) are reported in Figs. 16 ▸(*c*) and 16 ▸(*d*). At the lower strain rate, 10 s^−1^, the system is only partially ordered and the oriented h.c.p. layered structure coexists with randomly oriented structures that yield an isotropic peak-like scattering feature at *Q* ≃ 0.035 Å^−1^. At a strain rate of 100 s^−1^ the system may be considered to be in a fully ordered state and only spots are observed in the two-dimensional pattern, which produce distinct peaks in the averaged profiles over different angular sectors, as shown in Fig. 16 ▸(*d*).

**Figure 18:**
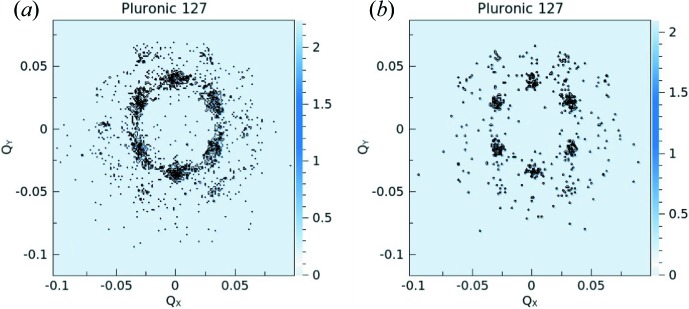
Two-dimensional scattering patterns from the Pluronic F127 system in D_2_O under oscillatory shearing with ω = 1 rad s^−1^ and strain rates 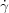 of (*a*) 10 s^−1^ and (*b*) 100 s^−1^, collected in the position of the maximum strain amplitude with a time resolution of 1.7 ms delivered by the chopper. The data measured in event mode were synchronized with the rheometer cycles in a post-acquisition SANS procedure.

The best time resolution which can be achieved with the chopper settings adjusted to this configuration (frequency *f*_chopper_ = 32.9 Hz and a slit opening of 20°) is 1.7 ms. It can be reduced to as low as 0.5 ms by further closing the slit opening. In order to check the instrument capability at such short time resolutions, we analysed the data within one rheometer cycle binned in 3695 channels each 1.7 ms wide. Fig. 18 ▸ displays the two-dimensional scattering patterns collected in each time slot in the position of the maximum strain amplitude. Although the intensity seems quite poor, the morphological changes experienced by the sample under the two different strain rates are visible. The averaged data over different angular sectors (Fig. S6 in the supporting information) show similar features to those observed in Figs. 16 ▸(*c*) and 16 ▸(*d*). The data statistics can be improved by increasing the total acquisition time.

### 4.5. Biological samples   

The characterization of single proteins in buffer solution is a challenging experiment since it involves low concentrations and low stability of the sample over time. A test experiment was done on streptavidin solutions at protein concentrations of 0.5 and 1.02 mg ml^−1^ involving two detection distances, *L*_D_ = 1.5 and 4 m, and with an acquisition time of 1800 s for the protein in buffer solution, the buffer solution, the empty cell and blocked beam measurements at every detection distance. The buffer solution was deuterated and all samples were measured in 2 mm path quartz cells with a beam size of 1 cm^2^. The counting rates involved in the experiments were about 160 kHz at *L*_D_ = 1.5 m and 20 kHz at *L*_D_ = 4 m, with detector sensitivity and absolute calibration carried out with a Plexiglas sample at 500 and 55 kHz, respectively. The collimation length was *L*_C_ = 4 m, the opening size of the entrance aperture being 50 × 50 mm. Recombinant streptavidin was obtained commercially (ProSpec-Tany TechnoGene Ltd, Israel). Protein solutions of 0.5 and 1.0 mg ml^−1^ in heavy water buffer (25 m*M* Tris–HCl, 120 m*M* NaCl, 5 m*M* KCl, 3 m*M* MgCl, 99.9% atom D, pH 7.4) were measured on KWS-2. Streptavidin forms a homotetramer in solution with a theoretical mass of 53.6 kDa, as calculated from the amino acid sequence.

**Figure 19:**
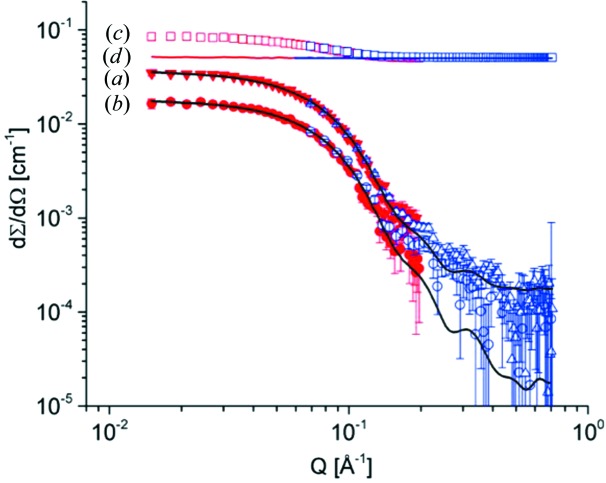
SANS patterns from streptavidin protein in buffer solution. The lower curves show the protein scattering at concentrations of (*a*) 1.02 mg ml^−1^ (triangles) and (*b*) 0.5 mg ml^−1^ (circles) after the correction for the scattering contribution from the buffer had been applied. The lines represent the fits to the experimental data according to models (see text). The upper data depict the one-dimensional corrected (empty cell, blocked beam and detector sensitivity) and calibrated intensity, (*c*) from the protein (1.02 mg ml^−1^) in buffer solution (open squares) and (*d*) from the buffer solution (line). The colours distinguish data measured at two detection distances.

The contribution of the D_2_O buffer was subtracted from the recorded SANS intensities of the protein solutions. Fig. 19 ▸ presents the corrected and calibrated one-dimensional data from the sample at 1.02 mg ml^−1^ in buffer solution and from the buffer solution, together with the protein contribution at the two concentrations after the correction for the buffer contribution had been applied.

Theoretical SANS curves were calculated on the basis of the known crystal structure (PDB code 1n4j) and fitted to the experimental SANS data using the program *CRYSON* (Svergun *et al.*, 1998 ▸). Guinier radii were calculated from the initial slope of a plot of ln*I*(*Q*) *versus Q* ^2^ using the program *PRIMUS* (Konarev *et al.*, 2003 ▸). The fits to the experimental data yielded radii of gyration *R*_g_ = 21.4 ± 0.08 Å and *R*_g_ = 21.8 ± 0.07 Å for the streptavidin in 0.5 and 1.02 mg ml^−1^ solutions, respectively. The calculated radius of gyration from the crystal structure is *R*_g_ = 21.9 Å.

In spite of the short acquisition time, good quality data were obtained for the 1.02 mg ml^−1^ protein solution. For the lower concentration, the data collected at high *Q* are very noisy. At this concentration, longer acquisition times are required in order to obtain better quality data in such a real experimental investigation.

The data were not corrected for the incoherent scattering from the protein. This is a very special correction which, for an unambiguous characterization of the protein morphology, would require measurements using polarized neutrons and polarization analysis. In this way the incoherent free signals from the protein buffer solution and from the buffer solution can be acquired separately from the *Q*-independent in­coherent background (Salhi *et al.*, 2017 ▸; Chen *et al.*, 2017 ▸). The use of a ^3^He spin analyser, as is planned to be used in the near future on KWS-2, should take care of a proper treatment of the inelastic scattering from protonated components in the sample, as reported by Chen *et al.* (2017 ▸). The inelastic scattering effects occurring in the case of incoherently scattering systems, such as biological macromolecules in a partially or fully protonated buffer solution which achieves special contrast-matching conditions and a physiological state of the system, induce a shift in neutron energy from longer to shorter wavelengths (Ghosh & Rennie, 1990 ▸; Rennie & Heenan, 1993 ▸; Do *et al.*, 2014 ▸). Consequently, a large portion of the neutrons are scattered by the sample with a gain in energy that corresponds to a wavelength distribution which is centred at around 1.5 Å (Ghosh & Rennie, 1990 ▸). Therefore, owing to the strong wavelength dependence of the ^3^He analyser, the polarizing efficiency and transmission for the inelastic part are different from those for the elastic part. Thus, the inelastic scattering from hydrogenated materials makes separation of the coherent and incoherent scattering by the usual SANS polarization analysis method incorrect. The use of TOF data acquisition for the correct interpretation of elastically scattered data is necessary when using spin analysis for the separation of the coherent signal from incoherent background for soft-matter and biological systems (Chen *et al.*, 2017 ▸). Although KWS-2 is not a dedicated TOF instrument, the inelastic scattering from highly protonated samples could be observed in special cases using the TOF data-acquisition mode for long detection distances and with long neutron wavelengths (Fig. S7 in the supporting information). Results collected with the old detection system on H_2_O samples for different sample thicknesses have shown that, even for thin samples (0.5 mm), the inelastic scattered neutrons represent an important fraction of the spectrum. The old scintillation detector had an efficiency of about 65% for neutron wavelengths in the range of 1–2 Å. Moreover, in the case of biological samples, the use of a sample thickness of about 1 mm is required owing to intensity needs. This makes it necessary to involve TOF data acquisition in future polarization analysis experiments on KWS-2 (Salhi *et al.*, 2017 ▸). For this purpose, further developments at the sample position are needed, including the installation of a dedicated mini chopper to enable proper TOF acquisition of data at different *L*_D_ and λ in order to properly detect the elastic scattering from such samples.

## 5. Conclusions   

A new fast detection system has been installed on the high-intensity SANS diffractometer KWS-2 operated by the JCNS at MLZ, Garching, Germany. The detector is composed of 18 eight-pack modules of ^3^He tubes that work independently of one another (each unit has its own processor and electronics). The main features of the detector obtained during performance characterization are summarized in Table 1 ▸. The new detector became operational for the user programme on KWS-2 in the second half of 2015. Research topics in the fields of soft matter and biophysics are already benefitting from the improved detection performance.

## Supplementary Material

Additional figures. DOI: 10.1107/S1600576718004132/uj5002sup1.pdf

## Figures and Tables

**Table 1:** Measured parameters of the new ^3^He detection system on KWS-2

Parameter	Whole system	Tube or eight-pack	Old detector
Efficiency (5 Å)	75%	85% (tube)	95%
Count rate for 10% dead time	6 MHz; up to 3 MHz for reliable corrections	50 kHz (tube)	100 kHz
Position resolution	<8 mm	<8 mm	∼7 mm
Position stability (r.m.s.)	<0.06 mm (eight-pack prototype)	<0.06 mm (eight-pack prototype)	
Position accuracy/linearity	1 mm	1 mm	
Pixel rate stability	<0.75%; within statistical uncertainty	<0.4%; within statistical uncertainty	
Dynamic *Q* range (*Q*_max_/*Q*_min_)	15		11
