# Supplementary material for: The high-intensity option of the SANS diffractometer KWS-2 at JCNS – characterization and performance of the new multi-megahertz detection system
Source: J Appl Crystallogr. 2018 Mar 28;51(Pt 2):323–36. doi: 10.1107/S1600576718004132 (PMC5884387; doi:10.1107/S1600576718004132)
Supplement: Supplementary file 1 [file j-51-00323-sup1.pdf]

JOURNAL OF APPLIED CRYSTALLOGRAPHY

Volume 51 (2018)

Supporting information for article:

The high-intensity option of the SANS diffractometer KWS-2 at JCNS – characterization and performance of the new multi-megahertz detection system

Judith Elizabeth Houston, Georg Brandl, Matthias Drochner, Günter Kemmerling, Ralf Engels, Aristeidis Papagiannopoulos, Mona Sarter, Andreas Stadler and Aurel Radulescu

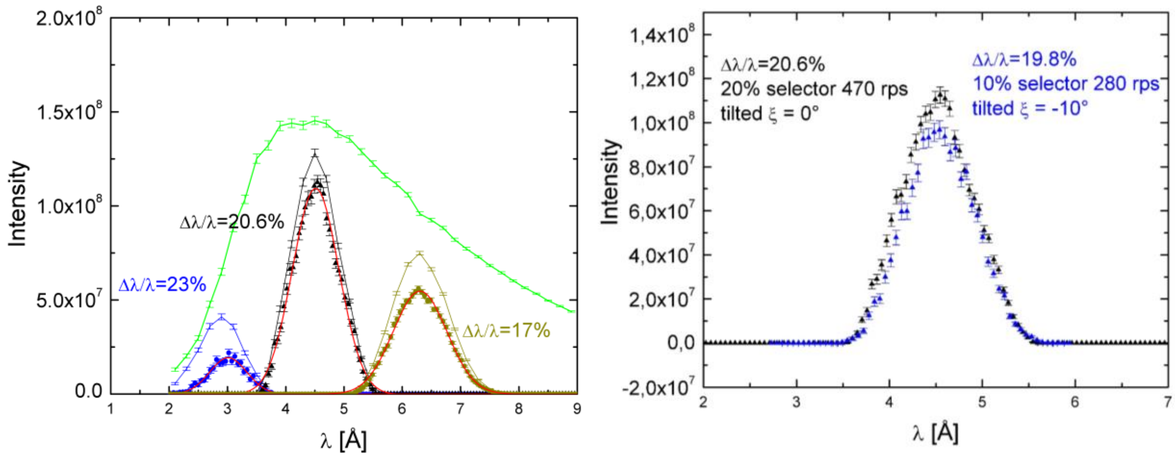

Figure S1 Results of McStas simulations of the wavelength distribution and intensity at different positions along the neutron guide system and instrument KWS-2. The left plot presents the case of the white beam before the velocity selector (green line) and the monochromatic beam immediately after the velocity selector 1 (32 blades) and in front of the collimation base for a rotating at a speed of the selector of 28200 r min^{-1}. The three Gaussian curves correspond to different tilt angles (blue: -10°; black: 0°; brown: +10°) of the selector with respect to the beam axis. The right plot shows the results at the sample position when two different selectors are used at different speeds and in different tilting positions with respect to the beam axis so that they provide a similar wavelength spread of the monochromatic beam.

Selector 1 (black symbols) is an ASTRIUM velocity selector with 32 blades that provide a nominal wavelength spread of 20%. Selector 2 (blue symbols) is an ASTRIUM velocity selector with 64 blades that provide a nominal wavelength spread of 10%.

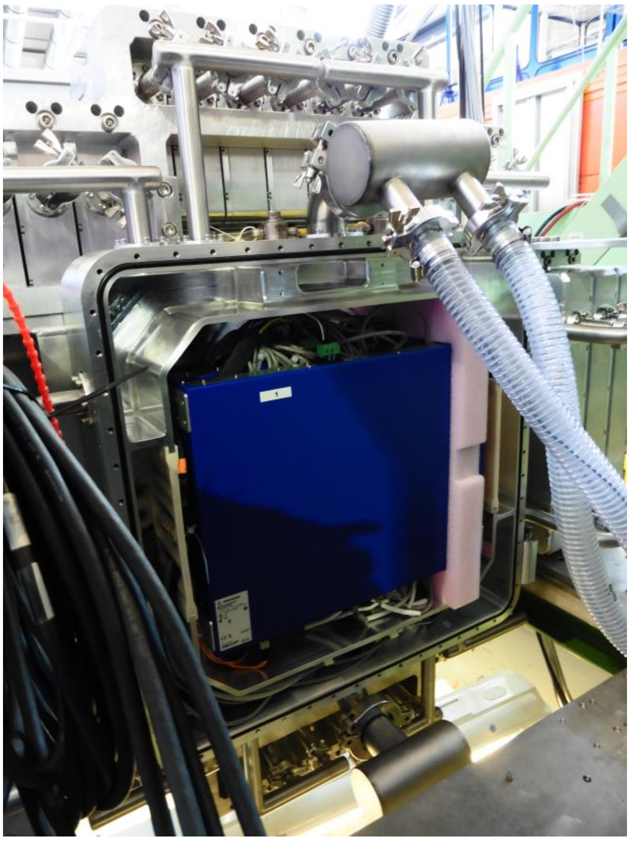

Figure S2 View of the detector from the rear: the air box (open in this photo, the cover is removed), the internal switches (Hirschmann) and the exhaust airline with the power and signal cables through the hoses can be seen. The photo was taken during the assembling phase of the new detector on KWS-2.

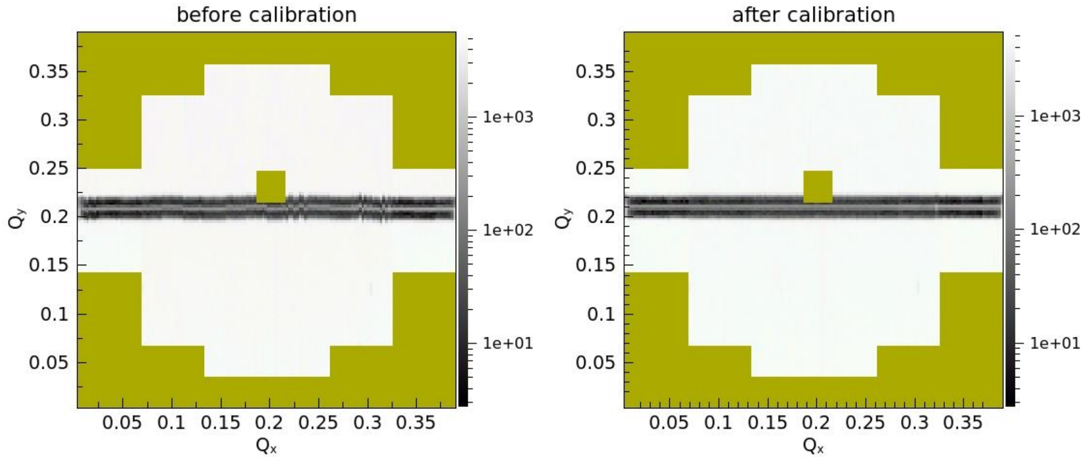

Figure S3 The scattering patterns from the Plexiglas sample, which provides a homogeneous scattering over the whole active area of the detector, taken before (left) and after the position calibration of the detector. For this operation a set of Cd plates defining a narrow 2 mm slit were mounted in front of the detector.

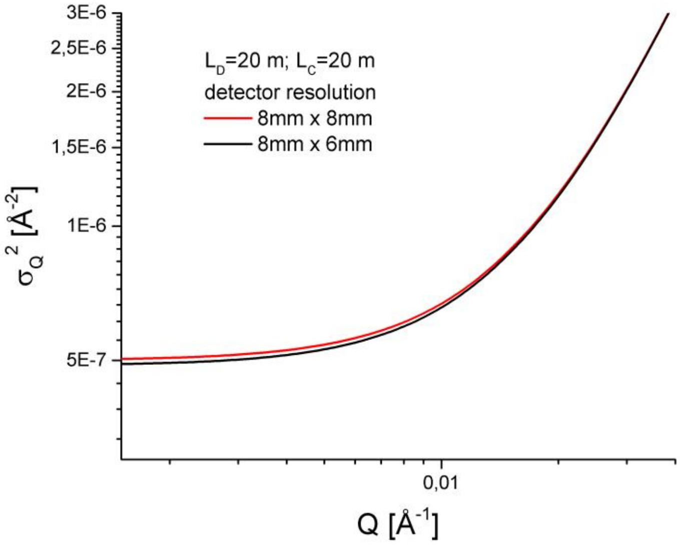

Figure S4 The minor effect of the detector resolution to the total *Q* distribution σ_Q^2; a specific experimental configuration on KWS-2 was considered: λ= 5 Å, L_D = 20 m, R_E = 16.9 mm, R_S = 5.64 mm, L_C = 20 m and wavelength spread $\Delta\lambda/\lambda$=10%. The curves were calculated for a detector resolution of 8mm x 6mm (black) and 8mm x 8mm (red), respectively. By taking into account that the placement of the short ^{3}He tubes which show a better resolution corresponds to the high *Q* scattering angles, there is no effect of the slightly better tube resolution at the lateral sides of the detector on the quality of data. The resolution for the data measured with the short tubes is dominated by the wavelength distribution.

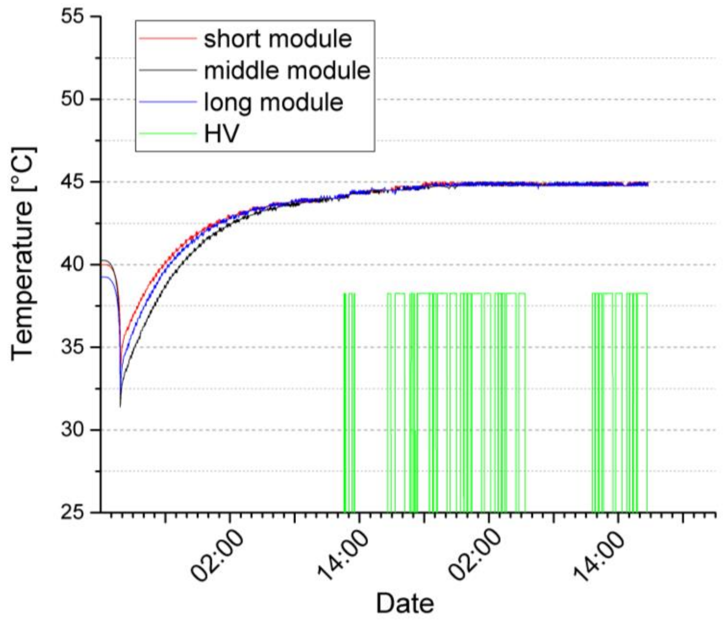

Figure S5 The temperature on the processor boards of three eight-packs collected over a long time period after turning on the operation voltage of the electronics well before the routine operation of the instrument. The status of the high voltage (HV) on the tubes is shown by the green line, with the upper flank meaning that the HV is on. During the measurements the temperature on the boards stays constant (about 45°C) regardless of the status of the HV.

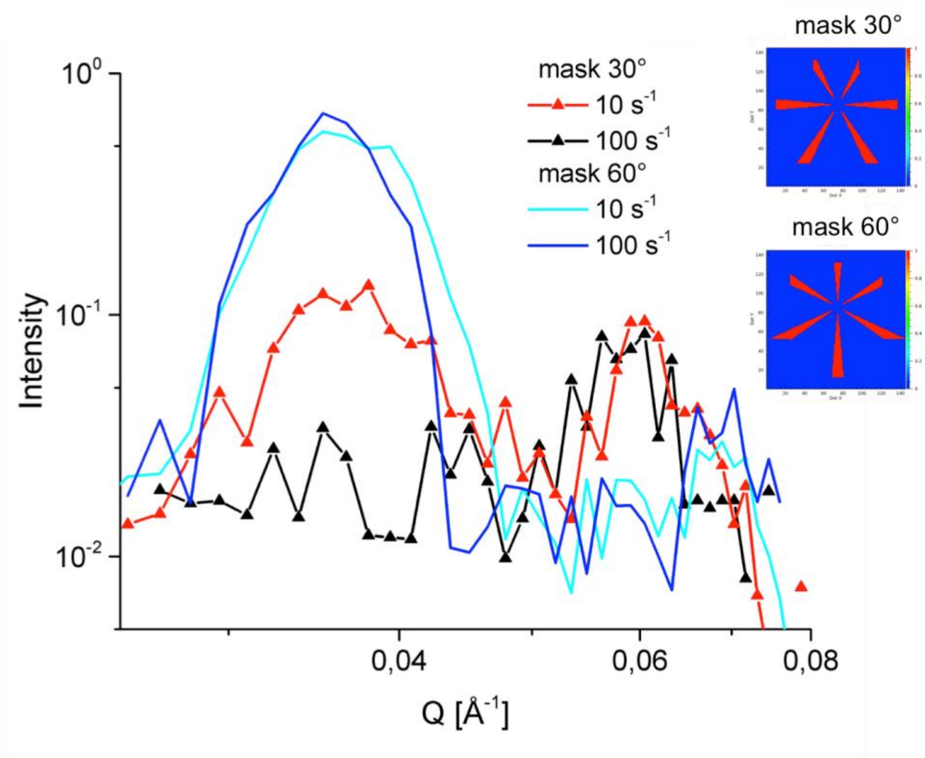

Figure S6 One-dimensional scattering intensity obtained after averaging the data in Fig. 14 over different angular sectors as shown in the insets.

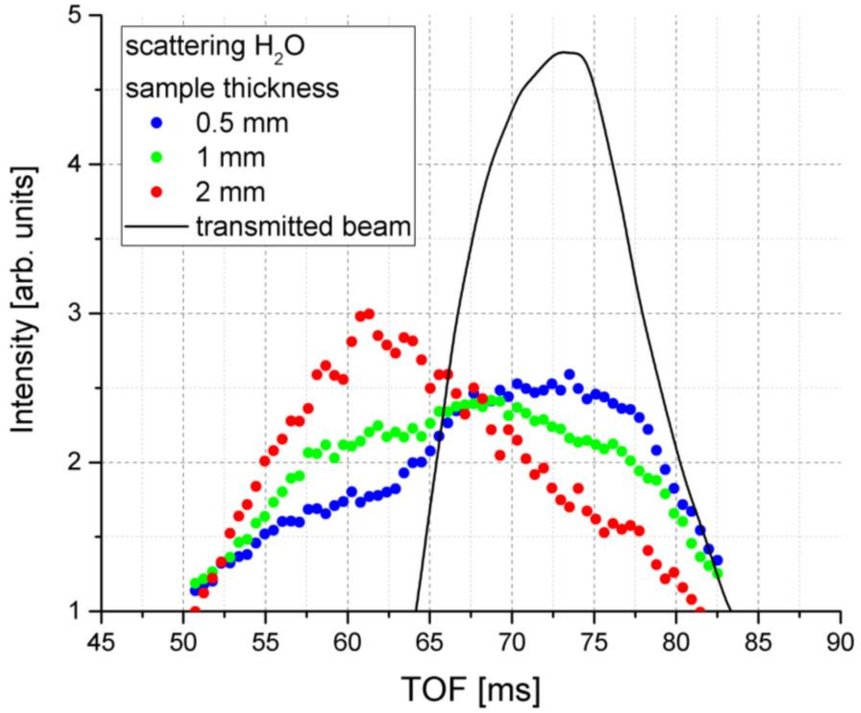

Figure S7 The scattered (dots) and transmitted beam from samples of H_2O with different thickness collected in TOF mode with the old KWS-2 detector placed at L_D = 8 m. The measurements were carried out using neutrons of incoming wavelength λ = 10Å and with the chopper frequency of $f_{chopper}$ = 16.4 Hz. For this instrument configuration the elastically scattered neutrons are grouped in the TOF region around 74 ms, as indicated by the transmitted beam. The distribution of inelastic scattered neutrons with a gain in energy, thus shorter wavelengths than the incoming ones, is clearly visible for all three samples in the TOF region of 50-70 ms. The TOF of 57.92 ms corresponds to neutrons that are scattered from the sample to the detector with a wavelength of 2 Å. According to Ghosh & Rennie (1999) the broad inelastic scattering observed from H_2O between 1 Å and 5 Å can be separated into a distinctive librational mode at 1.1 Å and a broad Maxwellian envelope around 2.5 Å. Clearly, in order to resolve the structure and morphology of small biological macromolecules in highly protonated buffer by using SANS with polarisation analysis, a detailed TOF analysis of the scattered data is necessary in order to focus only on the elastically scattered patterns (see the main text).
